# Structural, optical, and electrical dynamics of Cs_3_Fe_2_Cl_9_: a lead-free triple perovskite candidate for advanced optoelectronics

**DOI:** 10.1039/d5ra07744c

**Published:** 2026-01-02

**Authors:** Moufida Krimi, Miftah Ali Ben Yazeed, Ahmed Ali Awhida, Regis Barillé, Abdallah Ben Rhaiem

**Affiliations:** a Laboratory LaSCOM, Faculty of Sciences of Sfax, University of Sfax BP1171 3000 Sfax Tunisia abdallahrhaiem@yahoo.fr; b Department of Physics, Faculty of Science, University of Alasmarya Islamic Zliten Libya; c Higher Institute of Engineering Technologies Zliten Libya; d Université d'Angers, CNRS, Moltech-Anjou, SFR Matrix F-49000 Angers France

## Abstract

Research on perovskites is increasingly concentrating on lead-free compounds due to their superior structural stability and lower biological toxicity compared to lead-based alternatives. Those compounds provide a safer and more sustainable path with improved durability, making them suitable for demanding technological uses. This work reports a detailed study of the structural, optical, and electrical properties of the lead-free triple perovskite Cs_3_Fe_2_Cl_9_. The material crystallizes in the hexagonal system with the space group *P*6_3_/*mmc*, as confirmed by X-ray diffraction. The refined lattice parameters indicate better crystallinity and structural strength. Optical absorption measurements show a strong response in the ultraviolet range, with an indirect band gap of about 2.8 eV and a low Urbach energy of 0.98 eV, suggesting a lower density of defect states. Infrared spectroscopy reveals vibrational features characteristic of bioctahedral groups, thereby confirming the structural architecture of our compound. Electrical testing through AC conductivity measurements reveals thermally stimulated charge carrier hopping with activation energy near 1.38 eV. Conductivity increases with temperature, showing a shift in the conduction mechanism from orientational localized polaron hopping (single polaron hopping) at lower temperatures to more effective translational movement at higher temperatures.

## Abstract 2

Research on perovskites is increasingly concentrating on lead-free compounds due to their superior structural stability and lower biological toxicity compared to lead-based alternatives.

## Graphical abstract

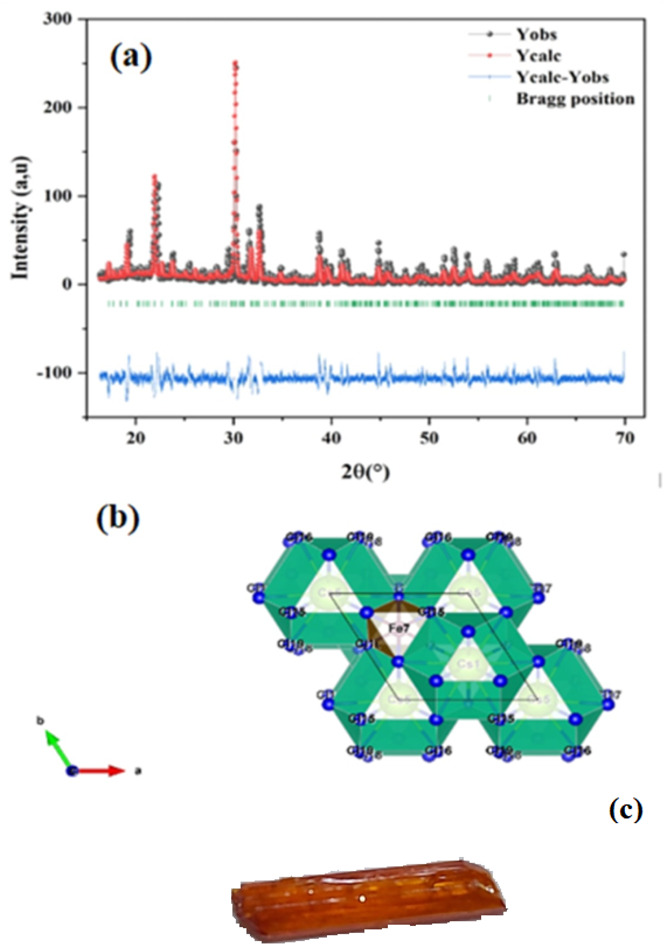

## 1 Introduction

Perovskites are among the most promising semiconductor materials for next-generation optoelectronics, having enabled remarkable advances in solar cells, light-emitting diodes, and photodetectors thanks to their excellent charge-carrier mobility, tunable band gaps, and solution-processable nature. However, the widespread adoption of conventional lead-based perovskites is hampered by the high environmental and health risks associated with lead. Recent environmental studies have demonstrated that Pb^2+^ ions can readily leach from degraded devices and migrate through soil and water systems under common environmental conditions such as moisture and temperature gradients posing long-term contamination hazards.^1–3^ These findings underscore the urgent need to eliminate lead at the source, particularly in consumer-oriented optoelectronic technologies that may be exposed to humidity, heat, or mechanical stress during their operational lifetime. This has accelerated the search for high-performance, lead-free alternatives especially among wide-band gap metal halide perovskites (WBG-MHPs, *E*_g_ > 2.3 eV) which are emerging as key enablers of UV-selective photodetection, scintillation, and radiation-hardened electronics. As highlighted in a recent comprehensive review,^4^ strategic compositional and dimensional engineering of WBG-MHPs unlocks unique functionalities for applications requiring visible-blind UV response or high-energy photon discrimination. For instance, Li *et al.*^5^ reported record X-ray sensitivity in lead-based WBG perovskites, intensifying the demand for non-toxic analogues with comparable performance. Moreover, Tian *et al.*^6^ recently discovered a triboluminescent response in WBG perovskite films under mechanical friction, revealing rich mechano-optoelectronic coupling and further expanding their potential in sensing and memory devices. Among lead-free candidates, vacancy-ordered triple perovskites with the general formula A_3_M_2_X_9_ (A = Cs^+^, Rb^+^; M = Sb^3+^, Bi^3+^; X = Cl^−^, Br^−^, I^−^) have garnered significant attention due to their structural robustness and favorable optoelectronic properties as evidenced by Cs_3_Sb_2_I_9_ (1.0% power conversion efficiency) and zero-dimensional Cs_3_Bi_2_I_9_ (photoluminescence quantum yield up to 99%).^7,8^ Nevertheless, not all A_3_M_2_X_9_ compositions are suited for light-emitting or photovoltaic applications. In particular, Fe^3+^-based derivatives despite their earth abundance, low cost, and negligible toxicity exhibit extremely weak or negligible photoluminescence owing to spin-forbidden d–d transitions and rapid non-radiative relaxation inherent to the high-spin d^5^ configuration of Fe^3+^.^9^

Crucially, magnetic studies confirm that Cs_3_Fe_2_Cl_9_ orders antiferromagnetically below *T*_N_ = 5.4 K, with dominant intra-dimer ferromagnetic coupling (*J*_1_ = +0.254 meV) outweighed by net antiferromagnetic inter-dimer interactions (Weiss temperature *θ*_CW_ ≈ −16 K).^10^ This dimer-based magnetic architecture, combined with its structural isolation, underpins rich spin-lattice physics and supports potential roles in cryogenic sensing and magnetodielectric applications despite its unsuitability for conventional photonic uses.

Consequently, Fe-containing halide perovskites are generally ill-suited for conventional photonic applications, prompting a shift toward exploring their potential in alternative functional roles such as radiation detection, magnetoelectrics, or dielectric system. This paradigm shift is strongly supported by recent findings: for instance, the structurally related compound Cs_3_Fe_2_Cl_5_·H_2_O exhibits intense X-ray scintillation with a light yield of ∼25 000 photons per MeV comparable to that of commercial scintillators.^11^ Moreover, the presence of Fe^3+^ imparts intrinsic paramagnetism and strong spin–lattice coupling, opening avenues for applications in magnetic sensing and spintronics devices. Although the thermoelectric performance of Cs_3_Fe_2_Cl_9_ has not yet been evaluated, its zero-dimensional (0D) crystal architecture and heavy chloride composition hallmarks of the A_3_M_2_X_9_ family are known to significantly suppress lattice thermal conductivity, a critical prerequisite for efficient thermoelectric, as evidenced in analogous 0D halide systems.^12^ Compounding these advantages, Cs_3_Fe_2_Cl_9_ also demonstrates notable chemical stability, a moderate effective atomic number (*Z*_eff_ ≈ 16.5), and inherent radiation hardness, all of which position it as a promising candidate for indirect radiation detection, particularly when integrated into composite or heterostructured device formats.

In this context, Cs_3_Fe_2_Cl_9_ a vacancy-ordered triple perovskite built from isolated [Fe_2_Cl_9_]^3−^ bioctahedral dimers separated by Cs^+^ cations emerges as a compelling platform to unlock these underexploited functionalities. Crystallizing in the hexagonal space group *P*6_3_/*mmc*, its structure features face-sharing FeCl_6_ octahedra that form dimeric units, effectively suppressing long-range electronic coupling while enhancing localized magnetic, vibrational, and dielectric responses.^13,14^ Our work addresses a critical gap in the field by providing the first comprehensive, multi-modal characterization of Cs_3_Fe_2_Cl_9_, encompassing its crystallographic integrity, UV-Vis optical behavior, FTIR vibrational signatures, and temperature-dependent electrical properties including complex permittivity, electric modulus, and AC conductivity. By establishing a rigorous structure–property framework, this study repositions Fe-based halide perovskites not as failed photonic emitters, but as earth-abundant, non-toxic, and multifunctional materials with tangible promise in next-generation applications ranging from radiation-hardened optoelectronics and magnetic sensors to energy-efficient dielectrics and beyond.

## 2 Experimental section

### 2.1. Synthesis

High-purity FeCl_3_·6H_2_O (99.99%, Sigma-Aldrich) and CsCl (99.999%, Sigma-Aldrich) were used as received without further purification. Crystals of Cs_3_Fe_2_Cl_9_ were synthesized *via* a hydrothermal method by first dissolving FeCl_3_·6H_2_O (0.526 g, 2 mmol) and CsCl (1.002 g, 6 mmol) separately in 37% hydrochloric acid to suppress Fe^3+^ hydrolysis and maintain a chloride-rich environment. The solutions were then combined and stirred at room temperature to form a homogeneous precursor mixture according to the stoichiometric reaction:3CsCl + 2 FeCl_3_·6H_2_O → Cs_3_Fe_2_Cl_9_ + 12H_2_O

The resulting solution was transferred into a Teflon-lined autoclave and heated at 180 °C for 12 hours to enable slow crystallization under autogenous pressure. After natural cooling to room temperature, intense red crystals of Cs_3_Fe_2_Cl_9_ characteristic of the dimeric [Fe_2_Cl_9_]^3−^ anion with face-sharing FeCl_6_ octahedra were collected, rinsed thoroughly with deionized water followed by ethanol, and subsequently dried at 60 °C under vacuum to remove residual surface moisture. The resulting phase-pure material was suitable for subsequent structural and physical characterization.

### 2.2. Characterization

X-ray powder diffraction (Cu Kα radiation, *λ* = 1.5406 Å, 15° ≤ 2*θ* ≤ 70°) confirmed compound purity at ambient temperature. UV-Vis spectroscopy (UV-3101PC, 200–800 nm) determined optical properties. Complex impedance spectroscopy (Solartron 1260, 0.1–106 Hz) was used to measure electrical properties over a temperature range of 393–453 K of 8 mm diameter, 1 mm thick pellets. Silver coating ensured reliable electrical contact.

## 3 X-ray result

**Fig. 1:**
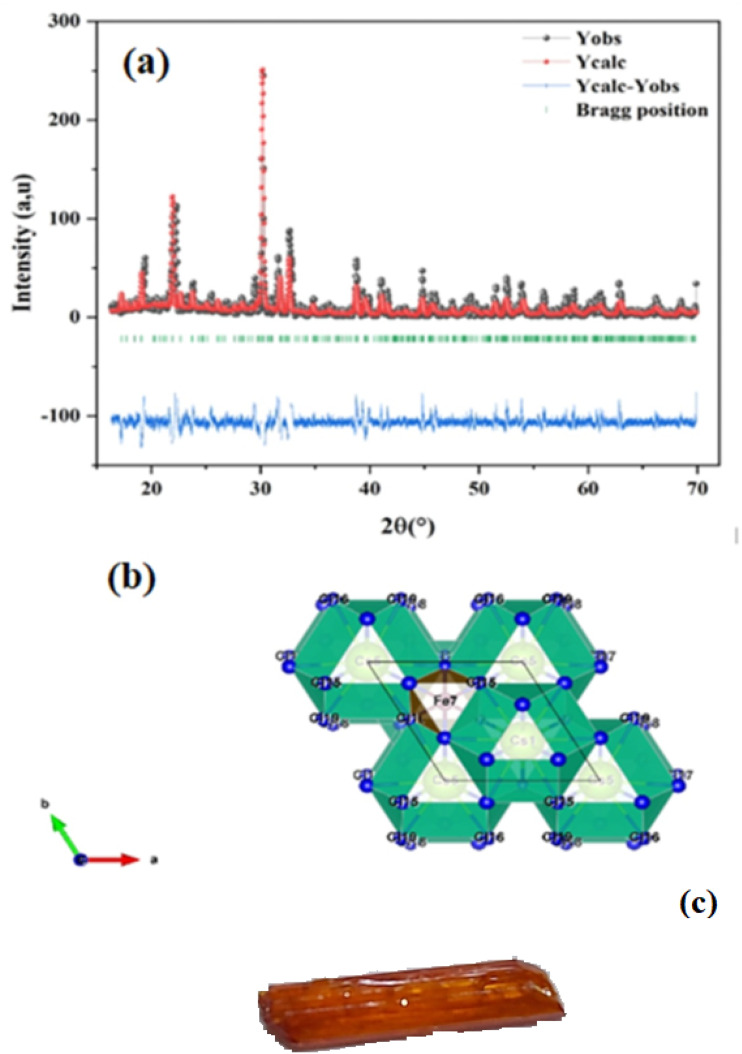
(a) The Rietveld refinement was performed on the X-ray crystal diffraction pattern of Cs_3_Fe_2_Cl_9_, (b) the crystal structure and (c) the obtained crystals of Cs_3_Fe_2_Cl_9_.

XRD data confirms Cs_3_Fe_2_Cl_9_'s hexagonal structure (space group *P*6_3_/*mmc*). Observed (*Y*_obs_) and calculated (*Y*_calc_) patterns show excellent agreement, supported by Bragg positions. The difference plot (*Y*_calc_ − *Y*_obs_) exhibits minimal deviation, with a good fit (*χ*^2^ = 3.5). Sharp, intense peaks indicate high crystallinity. The hexagonal arrangement reflects structural stability, crucial for understanding electronic properties and potential applications in optoelectronics. Accurate crystallographic parameters reveal ion interactions influencing physical and chemical behavior. The refined lattice parameters, *a* = [7.483] Å and *c* = [18.159] Å, further corroborate the layered structure inherent to the *P*6_3_/*mmc* space group. These dimensions dictate the Fe–Cl bond lengths and the inter-layer Cs–Cl distances, which are fundamental to the material's overall stability and its propensity to form excitons or facilitate charge transport. The narrow peak widths observed in the XRD pattern suggest minimal microstrain and a relatively large crystallite size, indicating a high degree of long-range order within the Cs_3_Fe_2_Cl_9_ lattice.^15^ This structural perfection is likely beneficial for achieving optimal performance in any device application. Ultimately, the combination of high crystallinity and well-defined hexagonal structure positions Cs_3_Fe_2_Cl_9_ as a promising candidate for exploration in diverse fields, ranging from light harvesting to spintronics. As shown in Fig. 1(c), the synthesized crystals are visually striking, presenting as transparent, amber-brown, well-faceted prismatic solids that attest to the high phase purity and structural perfection inferred from the diffraction analysis. This combination of structural precision and macroscopic crystal quality positions Cs_3_Fe_2_Cl_9_ as a compelling candidate for advanced applications in light harvesting, spintronics, and related fields.

The crystal structure of Cs_3_Fe_2_Cl_9_ adopts a hexagonal arrangement (space group *P*6_3_/*mmc*) and is built from face-sharing [FeCl_6_] octahedra, which form discrete [Fe_2_Cl_9_]^3−^ dimeric units the fundamental structural motif of this compound. In each octahedron, an Fe^3+^ ion occupies the central site, coordinated by six Cl^−^ ions at the vertices, establishing a typical octahedral geometry. The two [FeCl_6_] octahedra within each dimer share a common triangular face, resulting in a short Fe–Fe distance (∼2.9–3.1 A°), characteristic of face-sharing connectivity. These dimers are arranged in a hexagonal pattern along the *c*-axis and are isolated from one another by Cs^+^ cations, which reside in the interstitial spaces between the dimers. The large ionic radius of Cs^+^ ensures charge balance and enhances structural stability. The significant anisotropy in the lattice parameters *a* = 7.483 A° and *c* = 18.159 A° (with *c* ≈ 2.4*a*) reflects the layered nature of the structure, where Cs^+^ ions effectively separate the [Fe_2_Cl_9_]^3−^ layers along the *c*-direction. This zero-dimensional (0D) architecture suppresses long-range electronic coupling and underpins the material's localized electronic and magnetic behavior.^10^

## 4 UV-visible analysis

**Fig. 2:**
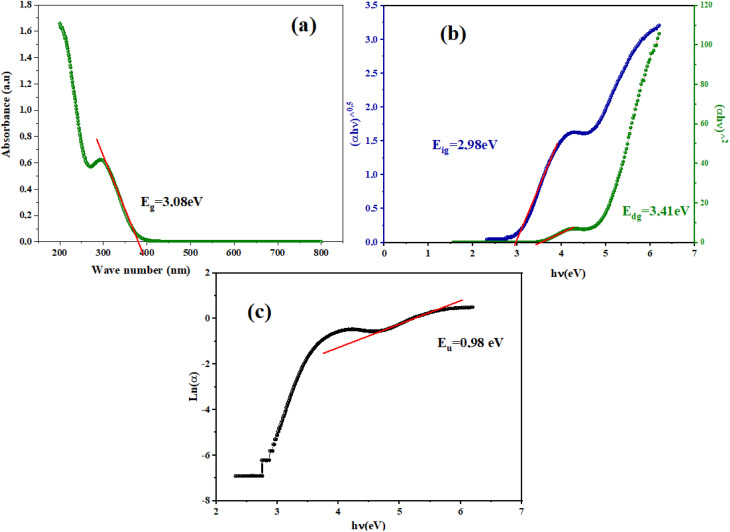
(a) Absorbance spectrum, (b) plot of (α*hν)^2^) and (α*hν)^1/2^) *versus* photon energy (*hν*), (c) ln(*α*) as a function of (*hν*) of Cs_3_Fe_2_Cl_9_ compound.

Room-temperature UV-Vis absorption spectra (298 K) of Cs_3_Fe_2_Cl_9_ reveals two distinct absorption regions. In the ultraviolet range (200–300 nm), the material exhibits strong absorption, with a prominent peak reaching approximately 1.65 a.u. around 200–220 nm. This intense UV absorption, which peaks between 200 and 220 nm, gradually diminishes as the wavelength increases toward 300 nm, reflecting high-energy electronic transitions characteristic of wide band gap semiconductors. Additionally, a secondary absorption feature is observed near 300 nm, with an absorbance of about 0.6 a.u., suggesting the presence of further electronic transitions (excitonic peak) within the material's band structure (d–d transition). The absorption edge is located between 350 and 400 nm, beyond which the absorbance drops sharply to near-zero values, indicating that the main optical transitions are complete by this wavelength. In the visible and near-infrared regions (400–800 nm), Cs_3_Fe_2_Cl_9_ displays minimal absorption, consistent with its wide band gap nature and its likely appearance as a light-colored or transparent material. The pronounced absorption in the UV region is attributed to charge transfer transitions involving Fe^2+^/Fe^3+^ centers and chloride ligands, as well as possible d–d transitions within the iron coordination environment. The negligible absorption in the visible range confirms that Cs_3_Fe_2_Cl_9_ is primarily UV-active, which may limit its direct use in visible-light photovoltaic applications unless modified by doping or structural engineering. As observed in Fig. 2(a), the second peak (around 300 nm, ∼0.6 a.u.) would correspond to the band-to-band transition (valence band to conduction band), while the intense first peak at 200–220 nm can be attributed to ligand-to-metal charge transfer (LMCT) transitions, where electrons are promoted from the filled chloride p-orbitals to the partially occupied d-orbitals of the iron centers (Fe^2+^/Fe^3+^), resulting in highly absorbing electronic transitions that are characteristic of transition metal halide complexes.^12^

Accurately determining the optical band gap of Cs_3_Fe_2_Cl_9_ is critical for optimizing its performance in UV photodetectors, photocatalytic applications, and scintillation devices. In this study, the following equation is used to calculate the band gap (*E*_g_) from the absorption spectra*αhν* = *B*(*hν* − *E*_g_)^*r*^where *B* is a constant, *α* = 2303 A e^−1^ and *r* is a constant, characterizing the nature of the optical transition between the valence band and the conduction band. Where *r* = 2 for an indirect allowed transition, *r* = 1/2 for an allowed direct transition. The optical analysis reveals Cs_3_Fe_2_Cl_9_ exhibits wide band gap semiconductor behavior with an indirect fundamental band gap of 2.98 eV, while the direct band gap transition occurs at a higher energy of 3.4 eV, reflecting the complex band structure typical of perovskite-related materials where the valence band maximum and conduction band minimum are located at different k-points in the Brillouin zone. The indirect nature is confirmed by the better linear fit in the (*αhν*)^1/2^*vs. hν* plot shown in Fig. 2(b). This wide band gap gives the material excellent UV absorption and impressive photo stability, but it comes with a trade-off, it can't effectively harvest visible light beyond 440 nm. This limitation steers the material away from conventional solar cell applications and toward more specialized uses like UV photodetectors or protective coatings.^13^ The relatively large band gap of Cs_3_Fe_2_Cl_9_ (∼2.98 eV) is consistent with the trend observed in A_3_B_2_X_9_ vacancy perovskites, where the replacement of heavy halogens (I, Br) with chlorine significantly increases the energy gap. For example, Cs_3_Bi_2_I_9_ has a gap of ∼2.1 eV, while its chlorine analogue Cs_3_Bi_2_Cl_9_ reaches ∼3.4 eV.^14^ In this context, the intermediate value of Cs_3_Fe_2_Cl_9_ reflects both the inductive effect of chlorine and the contribution of the 3d orbitals of Fe^3+^ to the band edges. While this puts Cs_3_Fe_2_Cl_9_ at a disadvantage for capturing visible sunlight compared to bromide or iodide variants, it actually makes the material quite attractive for applications that specifically need to work with high-energy UV photons.

In semiconductors and insulators, the absorption edge shows a sharp rise in absorption at photon energies near or above the band gap. Beyond this edge, the absorption spectrum exhibits an exponential increase known as the Urbach tail, which arises from localized states within the band gap caused by defects, impurities, or structural disorder.^15^ The Urbach tail energy, Eu, can be empirically determined using the Urbach–Martienssen law:
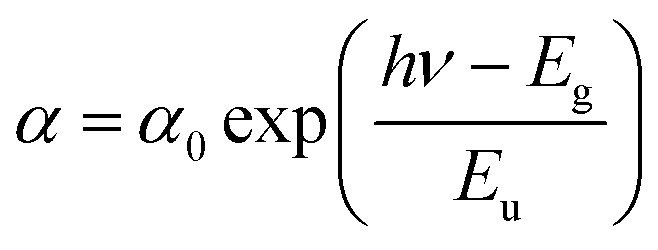
where *α*_0_ is a constant and *E*_u_ is the Urbach energy (eV). For our compound a significant amount of structural disorder is indicated by Urbach energy of 0.98 eV, which is roughly 35% of the band gap energy (2.98 eV). This value strongly indicates a high density of localized tail states within the band gap, which can impede efficient charge transport. It is also much higher than what is generally observed in high-quality crystalline semiconductors (where *E*_u_ < 0.1 eV). Grain boundary effects from the sample's polycrystalline nature, intrinsic lattice distortions associated with the zero-dimensional [Fe_2_Cl_9_]^3−^ dimeric structure, and possible stoichiometric variations or surface adsorbates added during hydrothermal synthesis are some of the likely causes of the high Urbach energy. The Urbach energy can be further analyzed using:
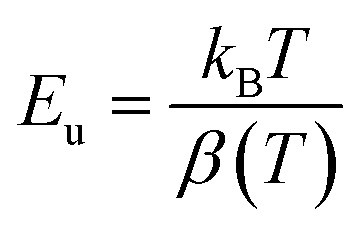
where *k*_B_ is Boltzmann's constant, *T* = 300 K is the absolute temperature, and *β*(*T*) quantifies the steepness of the absorption edge broadening due to electron–phonon interactions within the band gap.^16^ The parameter *β*(*T*) = 0.026, is indicative of the electron–phonon interaction strength (*E*_e–ph_) following the expression:
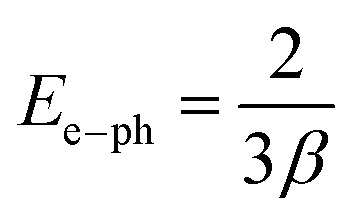


The electron–phonon interaction strength (*E*_e–ph_) is estimated to be approximately 25.64 eV.

## 5 Infra-red result

**Fig. 3:**
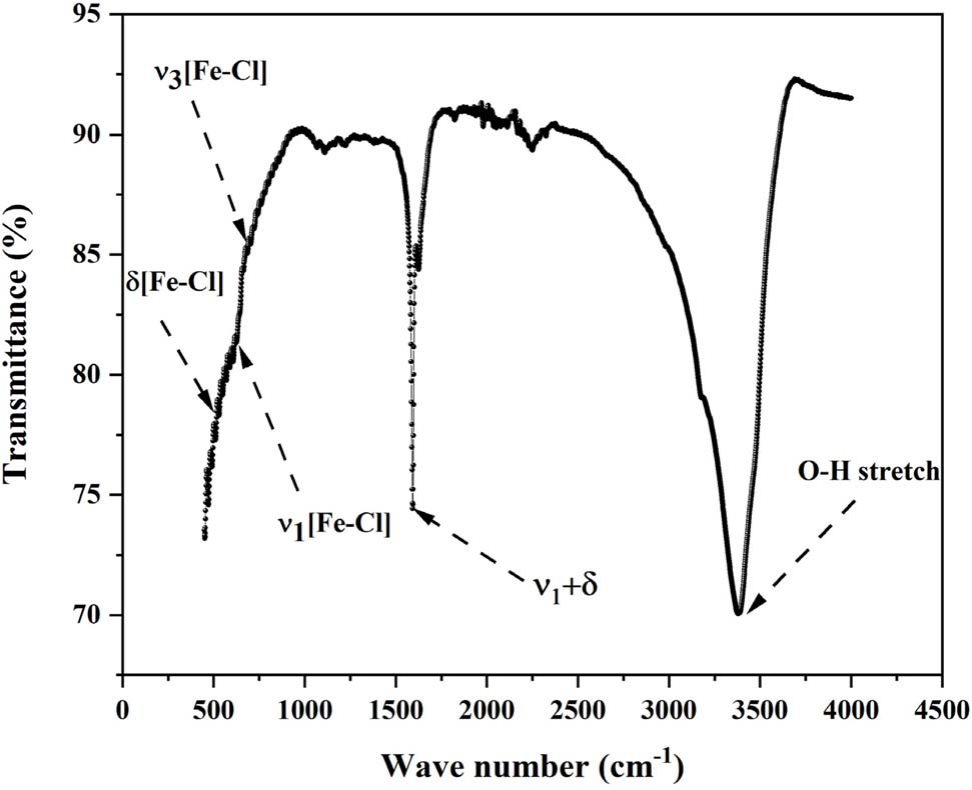
Infra red spectra of Cs_3_Fe_2_Cl_9_ perovskite.

Based on FTIR studies of structurally analogous Fe(iii) based chloride perovskites particularly Cs_2_FeCl_5_·H_2_O and CsFeCl_3_ the vibrational spectrum of Cs_3_Fe_2_Cl_9_ can be precisely assigned as follows (see Fig. 3). The strong, broad absorption in the 500–900 cm^−1^ region originates from internal vibrational modes of the isolated [Fe_2_Cl_9_]^3−^ bioctahedral units. Within this envelope, the peak at 720 cm^−1^ is assigned to the *ν*_3_ asymmetric stretching vibration of Fe–Cl bonds, the shoulder at 630 cm^−1^ corresponds to the symmetric stretch *ν*_1_(Fe–Cl), and a weaker band near 520 cm^−1^ is attributed to *δ*(Fe–Cl) bending (octahedral deformation) modes assignments fully consistent with reported data for FeCl_6_-based perovskites.^16^ A weak feature observed at 1520 cm^−1^ is identified as a first overtone or combination band (*e.g.*, *ν*_1_ + *δ*), indicative of anharmonic coupling between fundamental lattice modes.^17^ Finally, the intense and broad band centered at 3381 cm^−1^ is characteristic of O–H stretching vibrations from physisorbed water molecules; the absence of a corresponding H–O–H bending mode near 1630–1650 cm^−1^ confirms that the water is not chemically bound within the lattice but rather adsorbed on the surface. These IR findings validate the structural integrity of the material and confirm the presence of FeCl_6_ octahedral units.

## 6 Dielectric behavior

Dielectric studies are crucial for understanding the electrical properties of materials. By measuring the dielectric constant and loss factor over a range of frequencies and temperatures, researchers can gain insights into the polarization mechanisms, charge storage capabilities, and energy dissipation characteristics of a material. This information is essential for designing and optimizing capacitors, insulators, and other electronic devices. The dielectric constant demonstrates a material's dielectric response as follow:*ε*(*ω*) = *ε*′(*ω*) + *jε*″(*ω*)where *ε*′(*ω*) representing the real part of the complex dielectric constant refers to the energy stored outside *ε*″(*ω*) represents the imaginary part of the dielectric constant and reflects the energy dissipation of the applied electric field. The imaginary and real parts of the dielectric constant are related to the complex impedance by the following equations:
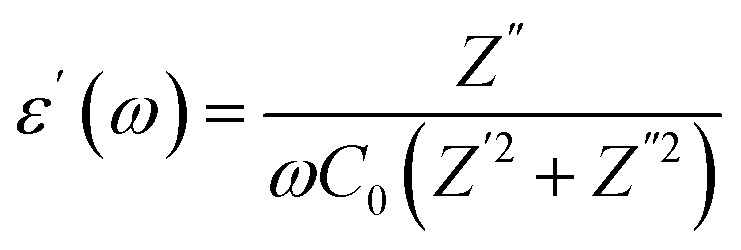

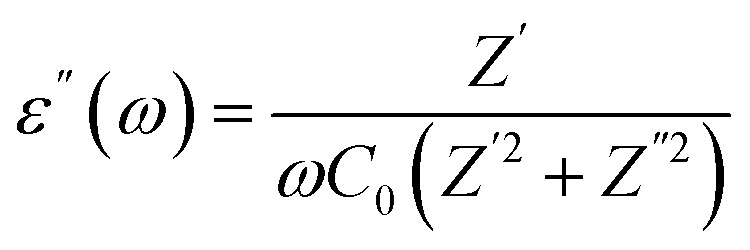
With *C*_0_ = (*ε*_0_*A*)/*d* (*C*_0_ is the capacity of free space while *ε*_0_ represents the permittivity of free space. *d* is the thickness of the pellet while *A* represents the surface of the electrode).^18^

**Fig. 4:**
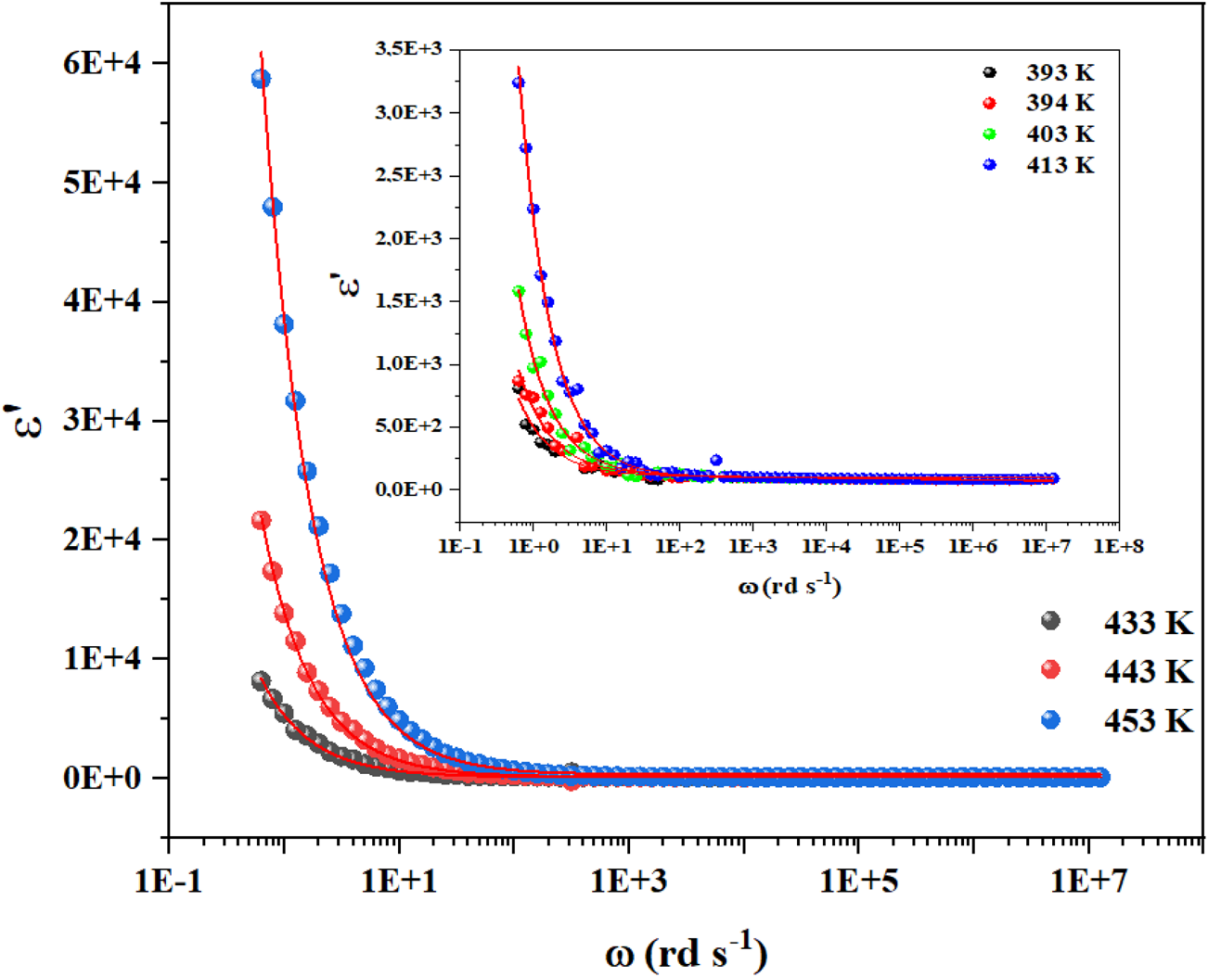
Influence of temperature on the real parts of the dielectric permittivity for Cs_3_Fe_2_Cl_9_ under specific range of frequency.

Fig. 4 displays the frequency dependence of the real part of the real and imaginary part of the dielectric permittivity (*ε*′) for Cs_3_Fe_2_Cl_9_ over a range of temperatures, with the main plot illustrating higher temperatures (433–453 K) and the insert focusing on lower temperatures (393–413 K).

**Fig. 5:**
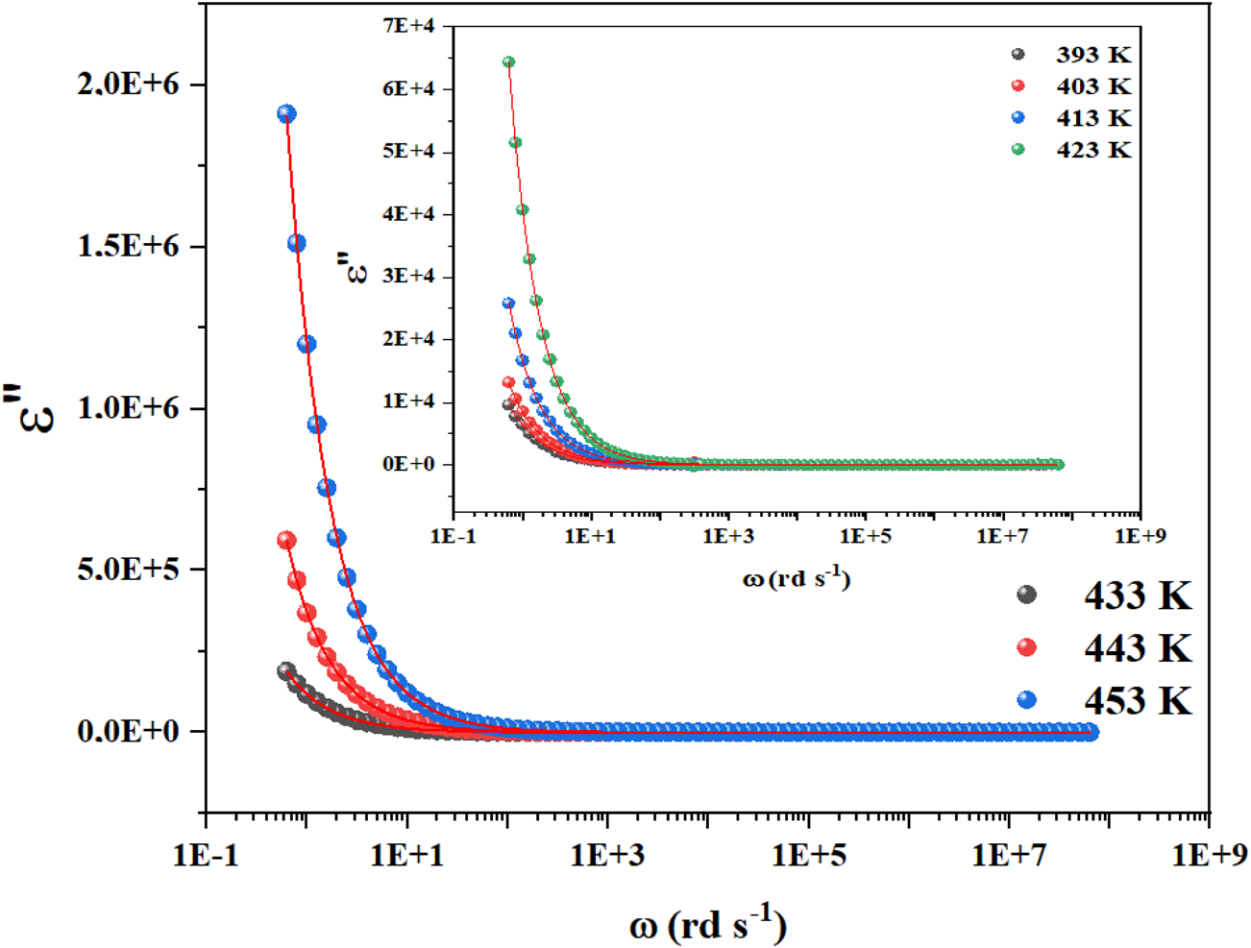
Influence of temperature on the imaginary parts of the dielectric permittivity for Cs_3_Fe_2_Cl_9_ under specific range of frequency.

Cs_3_Fe_2_Cl_9_'s permittivity (*ε*′ and *ε*″) varies with frequency and temperature due to multiple polarizations. *ε*′ decreases as frequency (*ω*) increases, peaking at low frequencies (*ω* down to 0.1 rad s^−1^) and stabilizing at higher frequencies (*ω* above 10^3^ rad s^−1^). *ε*″ also peaks at low frequencies before decreasing (Fig. 5). This low-frequency enhancement of *ε*′ and *ε*″ is mainly due to interfacial and dipolar polarization, which dominate ionic conduction and increase dielectric constant and loss.^19^ Interfacial effects in heterogeneous materials cause charge accumulation at boundaries. Above 10^4^ rad s^−1^, *ε*′ and *ε*″ become frequency-independent and decrease due to dipole and charge carrier lag. At high frequencies, less lossy ionic/electronic polarizations dominate. The increase of *ε*′ with temperature at low frequencies indicates thermally activated relaxations like ionic mobility, influencing polarization. This frequency–temperature interplay results in non-Debye relaxation in Cs_3_Fe_2_Cl_9_, highlighting interfacial polarization and thermal activation's importance in its dielectric properties.

A comparative analysis of the dielectric permittivity of Cs_3_Fe_2_Cl_9_ with related A_3_M_2_X_9_ halide perovskites reveals its distinctive behavior rooted in its zero-dimensional (0D) crystal architecture. At low frequencies (∼0.1 Hz) and elevated temperatures (393–453 K), Cs_3_Fe_2_Cl_9_ exhibits a high real permittivity (*ε*′ ≈ 10^3^–10^4^), significantly exceeding that of analogous bismuth- or antimony-based compounds such as Cs_3_Bi_2_I_9_ (*ε*′ ∼ 10^2^–10^3^) or Cs_3_Sb_2_I_9_ (*ε*′ ∼ 10^2^),^20,21^ which typically display lower values due to their more covalent bonding and reduced ionic mobility. This enhanced permittivity in Cs_3_Fe_2_Cl_9_ is primarily attributed to strong interfacial (Maxwell–Wagner) polarization and thermally activated polaron hopping, as confirmed by impedance and electric modulus analyses. In contrast, layered or 2D variants like Rb_3_Bi_2_I_9_ ^20^ show higher intrinsic conductivity but lower permittivity dispersion, reflecting more efficient inter-octahedral charge transport. The high *ε*′ of Cs_3_Fe_2_Cl_9_ though accompanied by substantial dielectric losses at low frequencies highlights its potential for applications in energy storage, UV-selective dielectrics, or radiation-hardened sensors, where interfacial effects and localized polarization are advantageous. This comparison underscores how structural dimensionality, halide identity (Cl^−^*vs.* I^−^/Br^−^), and the nature of the metal center (Fe^3+^*vs.* Bi^3+^/Sb^3+^) critically govern dielectric response in the A_3_M_2_X_9_ family.

Based on the provided information the modified Cole–Cole model was chosen for the modeling of the experimental data:
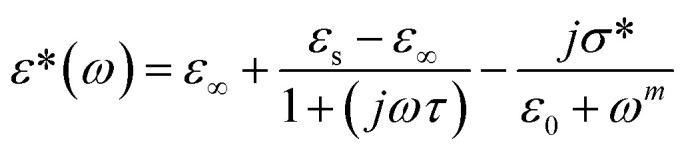
With σ* represents the sum of the conductivity of the free charge carriers (*σ*_fc_) and that of the space charge carriers (*σ*_sp_), *ε*_∞_ is the limit of the dielectric permittivity at high frequencies, while the limit at low frequencies presented by the term *ε*_s_. The parameter “*τ*” symbolizes the relaxation time, “*m*” indicates the frequency exponent and *β* is the modified Cole–Cole parameter which has a value between 0 and 1.^22^ The real and imaginary parts of the permittivity are adjusted respectively by the two following equation:
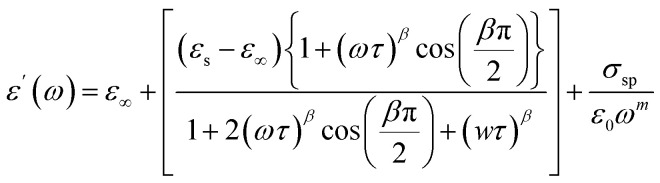

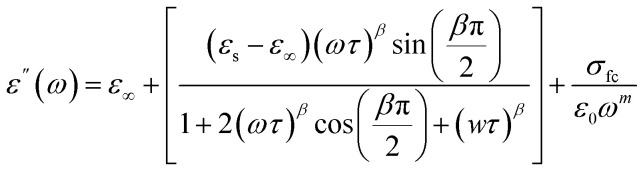


The previous equations yield agreement between experimental and theoretical data. The Ω^−1^ cm^−1^ resulted parameters from the adjustment of imaginary part of permittivity are listed in Table 1.

**Table 1:** The parameters *τ*, *α*, *m* and *σ*_fc_ evaluated from the fitting of the dielectric loss with the modified Cole–Cole model

Temperature (K)	*τ* (10^−4^ s)	*α*	*m*	*σ*_fc_ (10^−7^ Ω^−1^ cm^−1^)
393	2.68	0.11	0.18	110
403	8.71	0.17	0.23	32.8
413	5.22	0.31	0.13	10.5
423	4.09	0.92	0.17	3.53
433	0.13	0.16	0.14	1.1
443	6.16	0.12	0.22	0.70
453	1.6	0.42	0.15	0.41

The fitted parameters from the modified Cole–Cole model provide valuable insights into the dielectric relaxation and conduction mechanisms in the material. The relaxation time (*τ*) reflects the characteristic response time of dipolar or ionic polarization to the applied alternating electric field, generally decreasing with increasing temperature as charge carriers or dipoles reorient more rapidly due to enhanced thermal energy. The distribution parameter (*α*) quantifies the spread of relaxation times, with higher values indicating increasingly non-Debye, heterogeneous relaxation behavior; its increase at elevated temperatures suggests more complex microscopic environments, such as defects or grain boundaries, affecting polarization dynamics. The parameter *m*, representing the magnitude of dielectric loss associated with relaxation varies with temperature and suggests that the strength of polarization mechanisms peaks at moderate temperatures before declining, possibly due to increased carrier delocalization.^16^ Meanwhile, the conductivity-related parameter (*σ*_fc_) decreases with temperature, indicating a shift from localized hopping or interfacial conduction to more intrinsic charge transport and reduced space charge polarization. Collectively, these trends reveal a transition from slower, localized dipolar relaxation at low temperatures to faster, thermally activated, and more complex polarization processes at higher temperatures, reflecting the interplay between structural disorder and charge carrier dynamics. This comprehensive analysis underscores the material's behavior as governed by thermally activated ionic or polaronic motion and highlights the evolution from interfacial effects toward intrinsic dielectric responses with rising temperature, as effectively captured by the modified Cole–Cole model.^15^ To distinguish contributions in the material by unmasking the electrode effect, we use the electrical modulus, a valuable technique that offers insights into the relaxation dynamics and polarization mechanisms of materials, elucidating their response to an applied electric field across frequencies or temperatures.

**Fig. 6:**
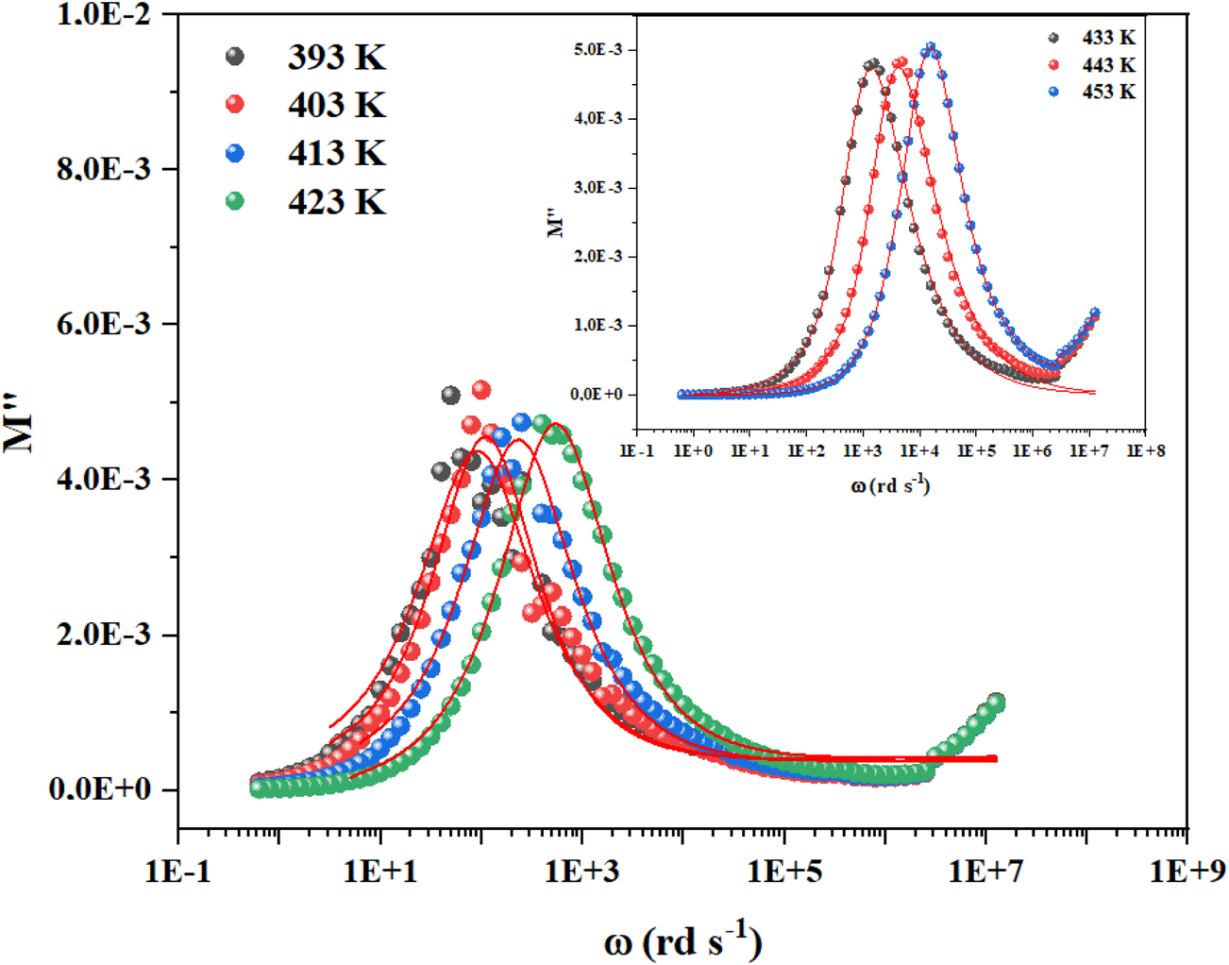
Frequency variation of the imaginary part of complex modulus for Cs_3_Fe_2_Cl_9_.

**Fig. 7:**
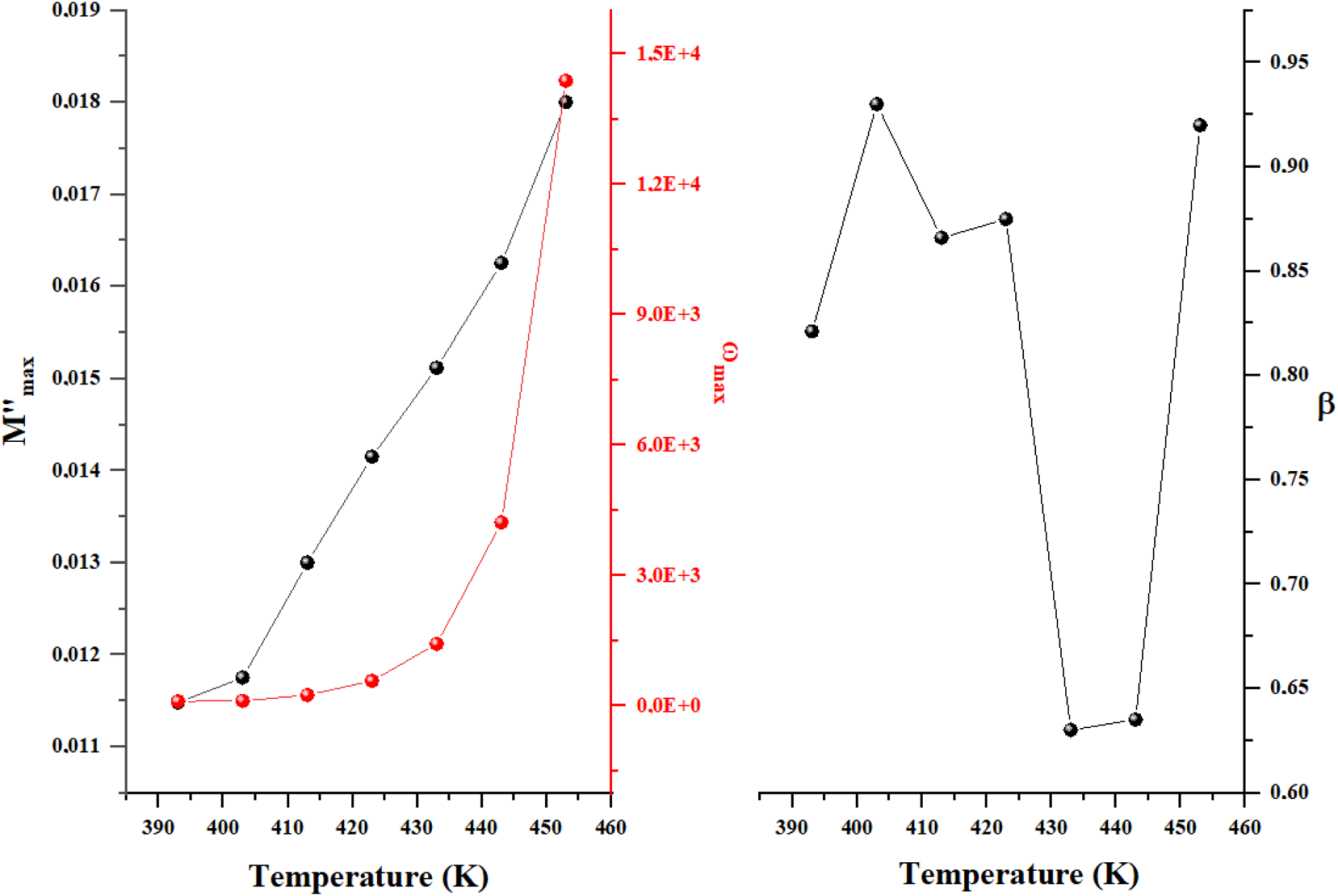
Temperature dependency of Bergman's parameter for Cs_3_Fe_2_Cl_9_.

The complex modulus is the reciprocal of complex permittivity and is expressed by the following expression (Fig. 7):
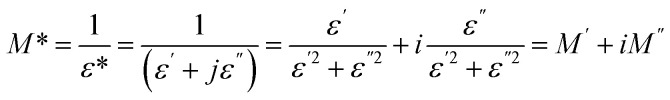


As shown in Fig. 6 the asymmetric peaks in the imaginary component of the electric modulus (*M*″) exhibit broader widths. Increasing temperature shifts the peak maxima to higher frequencies, a trend confirmed by Bergman's equation analysis:
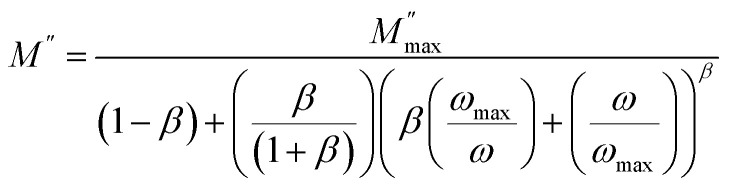
where *β* is the Kohlrausch parameter, which ranges from 0 to 1, and 
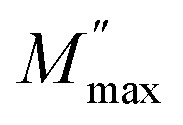
 is the maximum of the complex modulus corresponding to *ω*_max_.^16^ We have obtained a good agreement between theoretical model and experimental data, which allows us to represent the temperature dependency of the mentioned parameters as illustrated in Fig. 7:

The increase in the maximum of the imaginary part of the electric modulus 
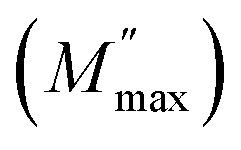
 with temperature indicates stronger relaxation strength due to enhanced charge carrier mobility, characteristic of thermally activated processes. The peak relaxation frequency (*ω*_max_) increases sharply above 430 K, signifying faster relaxation at higher temperatures. The shape parameter (*β*) shows a non-monotonic trend, but it is still under unity which confirms our model choice.^23^

## 7 AC conductivity

The AC electrical conductivity (*σ*_ac_) of Cs_3_Fe_2_Cl_9_ was measured as a function of angular frequency (*ω*) over a temperature range of 393 K to 453 K, revealing distinct behaviors influenced by structural and dynamic changes in the material. At all temperatures, the conductivity exhibits two characteristic regions: a low-frequency plateau corresponding to the DC conductivity (*σ*_dc_) and a high-frequency region where *σ*_ac_ increases with frequency, following a power-law behavior typical of hopping conduction mechanisms.^24^

This frequency-dependent behavior suggests that charge transport in Cs_3_Fe_2_Cl_9_ is dominated by localized charge carriers undergoing thermally activated hopping between states. The AC conductivity highlights a significant increase in conductivity with increasing temperature. This enhancement is consistent with thermally activated conduction, where higher temperatures provide sufficient energy for charge carriers to overcome potential barriers between localized states.

**Fig. 8:**
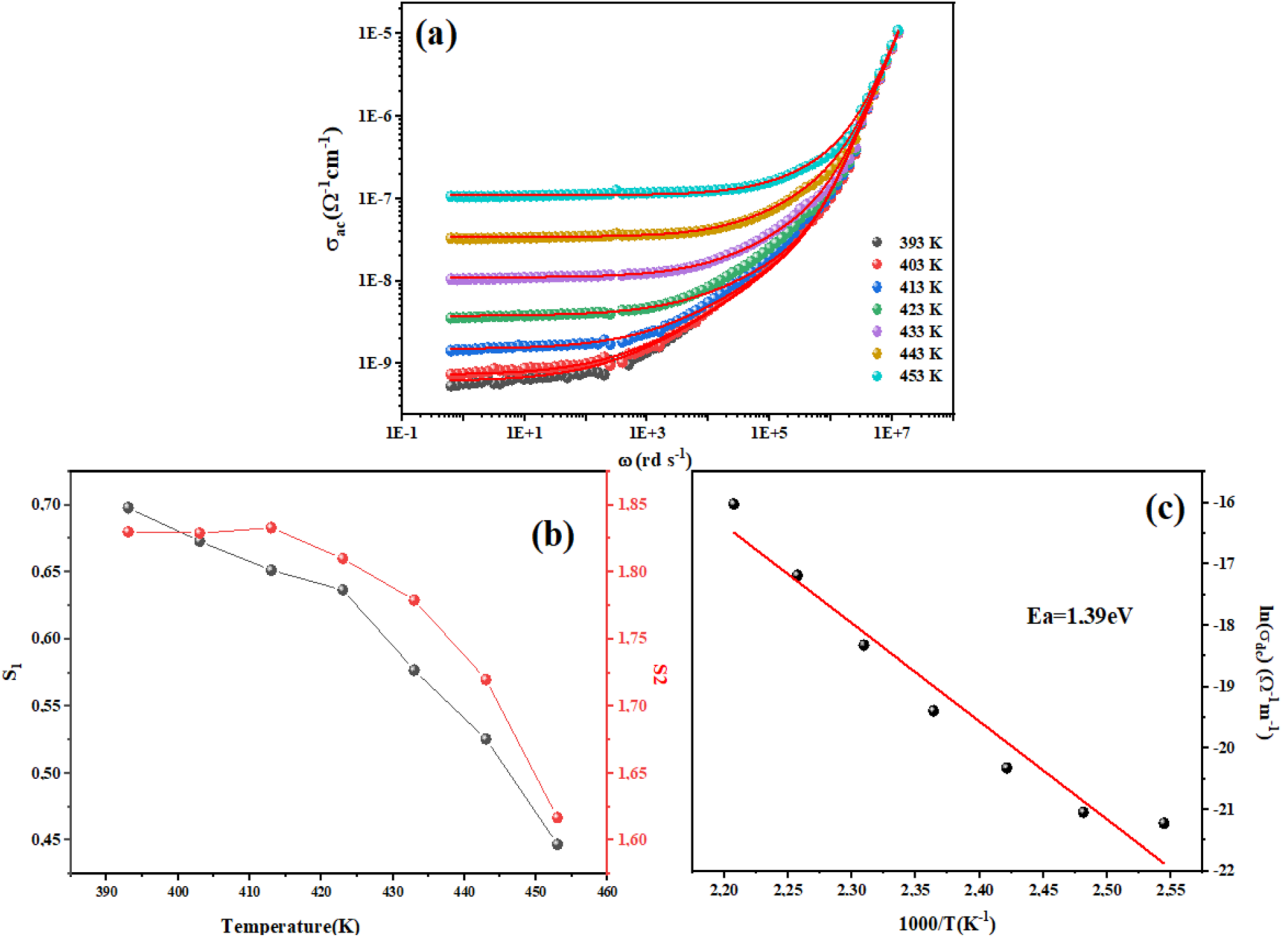
(a) Fitted curves of AC conductivity, (b) ln(*σ*_dc_) *vs.* 1000/*T*, (c) the exponent *s*_1_ and *s*_2_ of Cs_3_Fe_2_Cl_9_ compound.

The frequency dependency of the AC conductivity for Cs_3_Fe_2_Cl_9_is modeled by the double power law expression:*σ*_AC_ = *σ*_dc_ + *A*_1_*ω*^*s*_1_^ + *A*_2_*ω*^*s*_2_^where *σ*_dc_ is the pre-exponential factor, while *A*_1_ and *A*_2_ are the scaling coefficients, temperature-dependent parameters. The *s*_1_ term (with 0 < *s*_1_ < 1) dominates at low frequencies, representing the motion of short-range mobile ions. On the other hand, the *s*_2_ term (with 1 < *s*_2_ < 2) describes the high-frequency dispersion region, associated with localized hopping or reorientation motion.^16^Fig. 8(a) shows the AC conductivity of Cs_3_Fe_2_Cl_9_ between 393 K and 453 K as a function of frequency. The strong agreement between theoretical and experimental results validates the double power law expression.

The fit yields key parameters describing charge carrier motion and the conduction mechanism, including *σ*_dc_ and the exponent *s*. These parameters provide valuable insights into the material's electrical properties and can be used to predict its performance in various applications. Furthermore, the consistency between theory and experiment suggests that our model accurately captures the underlying physics of the charge transport process in this material. From the previous adjustment, the temperature dependence of *σ*_dc_ was obtained us depicted in Fig. 8(b). The data points align well with a straight line, indicating thermally activated conduction behavior that follows the Arrhenius law:^25^
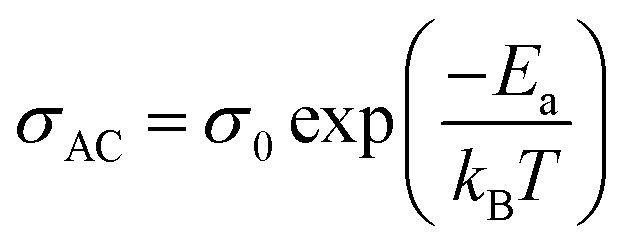
where *E*_a_ is the activation energy, *k*_B_ is Boltzmann's constant, and *T* is the absolute temperature. The linear fit yields a high correlation coefficient (*R*^2^ = 0.96), confirming the validity of the Arrhenius model for this system. The slope of the fitted line allows for the calculation of the activation energy, which is found to be 1.38 eV.

Fig. 8(c) illustrates the temperature dependence of the exponents *s*_1_ and *s*_2_, extracted from the AC conductivity data of Cs_3_Fe_2_Cl_9_ using the double power law model. The low-frequency exponent *s*_1_ decreases from approximately 0.70 at 393 K to 0.45 at 453 K. This trend is consistent with the Correlated Barrier Hopping (CBH) model, where charge transport occurs *via* thermally activated hopping of carriers over coulombic barriers between localized states.

In contrast, the high-frequency exponent *s*_2_ remains in the range of 1.60–1.83, which is characteristic of localized charge transport mechanisms. The slight decrease in *s*_2_ with temperature suggests that thermal activation enhances the overlap between localized states, promoting more efficient high-frequency conduction. Importantly, this behavior reflects charge carrier dynamics, not dipolar or orientational polarization, which are more appropriately analyzed through dielectric permittivity or electric modulus formalisms.^26^

Overall, the parallel decrease of both exponents with temperature reflects enhanced mobility and dynamic response of charge carriers, confirming that at low frequencies, conduction is governed by the CBH mechanism, while at high frequencies; localized relaxation remains the primary process.

### 7.1. The conduction mechanism study

**Fig. 9:**
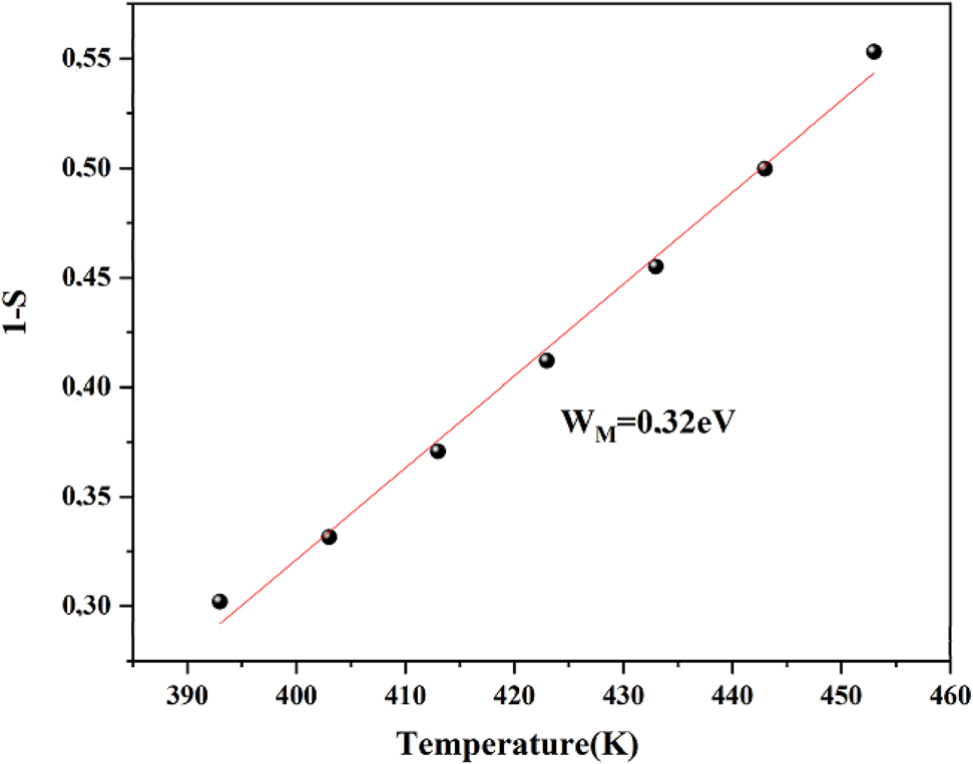
Variation of (1 − *s*) as a function of temperature for Cs_3_Fe_2_Cl_9_ compound.

The conductivity of the CBH (correlated barrier hopping) model is defined as follows:^27^
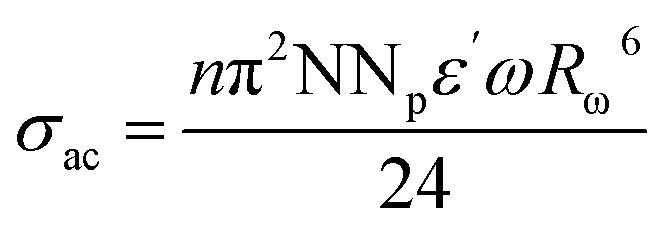
Here, *n* is the number of polarons involved in hopping, NN_p_ is proportional to the square of the localized states density, and *ε*′ is the dielectric constant at a fixed frequency. *R*_ω_ represents the jump distance, given by:
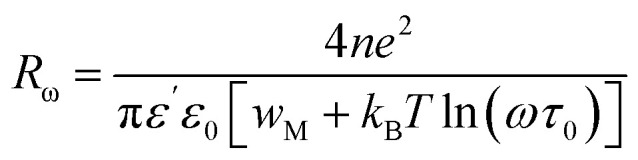
where *w*_M_ is the potential barrier height. Hopping can occur *via* a single polaron (single polaron hopping) or two polarons (two electrons hopping simultaneously between defects). The exponent “*s”* for this model is expressed as follow:
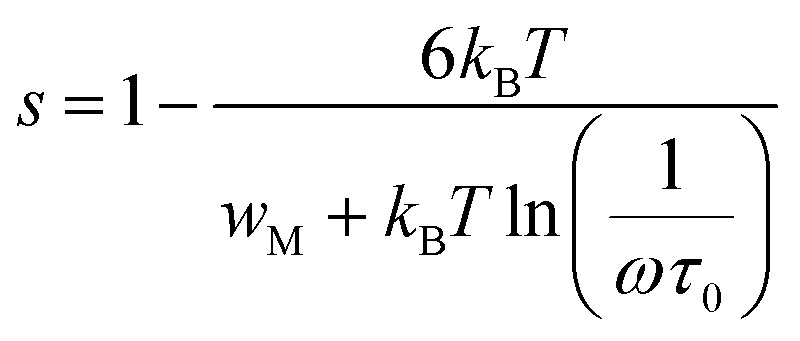


For large values of 
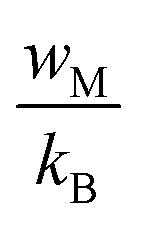
, the exponent *s* is expressed as follows:
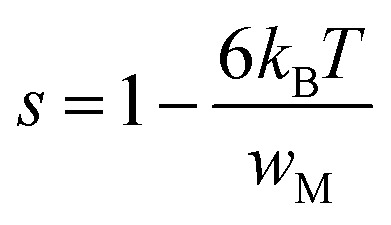


The relationship between *w*_M_ and the activation energy (*E*_a_) indicates the number of charge carriers involved in the jump: *w*_M_ = *E*_a_/2 suggests conduction *via* the correlated jump of two polarons, while *w*_M_ = *E*_a_/4 indicates a single polaron jump.^28,29^Fig. 9 shows the linear fit of the (1 − *s*) curve *versus* temperature.

**Fig. 10:**
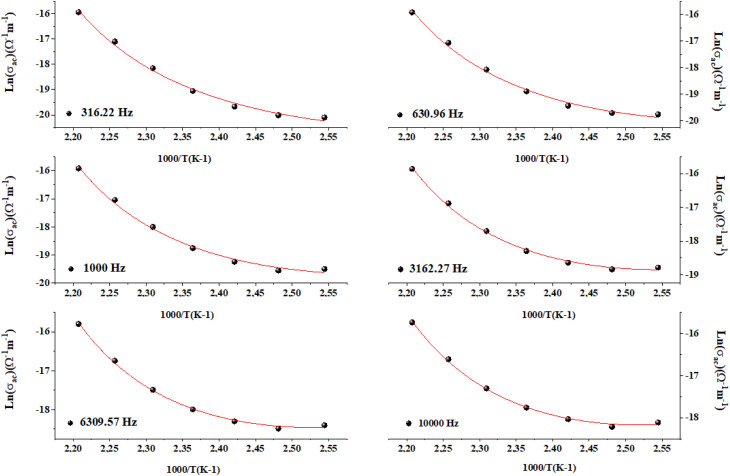
Temperature dependence of AC conductivity for the CBH model for Cs_3_Fe_2_Cl_9_.

The *w*_M_ value (0.32 eV) is about a quarter of the activation energy (1.38 eV), suggesting single polaron hopping which indicates a localized lattice distortion, facilitating efficient charge carrier transfer. This underscores the importance of electron–phonon interactions in conduction, elucidating the electrical properties of the compound. Ln(*σ*_ac_) adjustment *versus* (1000/*T*) aligns theoretical and experimental data, as shown in Fig. 10 validating our approach. This adjustment enabled us to determine several key parameters, as detailed in Table 2.

**Table 2:** Parameter resulting from CBH fit

Frequency (Hz)	*w*_M_ (eV)	Ueffect (eV)
316.22	0.93	−4.45
630.96	0.93	−6.52
1000	0.93	−7.81
3162.27	0.93	−8.42
6309.57	0.93	−9.88
10 000	0.93	−10.07

**Fig. 11:**
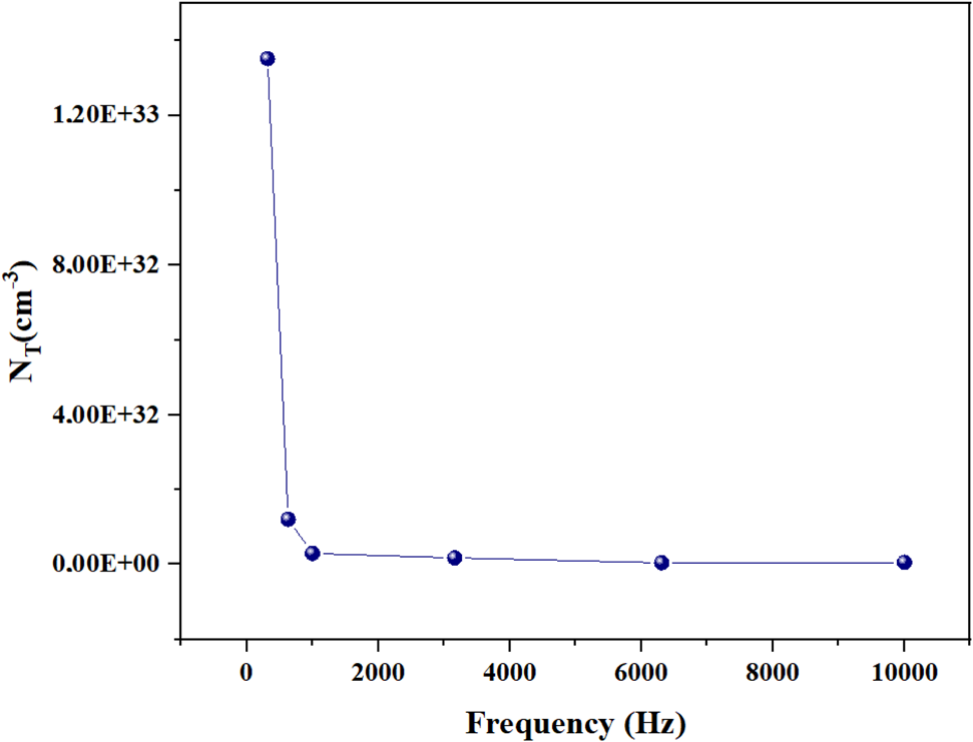
Frequency variation of the localized states density.

Additionally, we assessed how the density of localized states varies at different selected frequencies. The density of localized states reflects the number of electronic sites available for charge carriers. In single polaron hopping, the density of localized states decreases with increasing measurement frequency (Fig. 11).^30^ This is because, at higher frequencies, charge carriers have less time to hop between sites, effectively reducing the number of available sites they can interact with. This relationship between the density of localized states and frequency provides insights into the dynamics of charge transport in disordered materials.

The electrical transport properties of Cs_3_Fe_2_Cl_9_ characterized by a high activation energy (*E*_a_ = 1.38 eV) and a conduction mechanism dominated by single small polaron hopping, as confirmed by the Correlated Barrier Hopping (CBH) model are deeply rooted in its zero-dimensional (0D) crystal architecture, where isolated [Fe_2_Cl_9_]^3−^ bioctahedral dimers are separated by Cs^+^ cations. This structural motif strongly localizes charge carriers, particularly due to the high-spin d^5^ configuration of Fe^3+^, which suppresses band-like transport and enhances electron–phonon coupling. In contrast, the bromide analog Cs_3_Fe_2_Br_9_ exhibits a significantly narrower band gap (∼1.65 eV) and antiferromagnetic ordering below 13 K, reflecting stronger Fe–Br orbital overlap and reduced localization, though its conduction mechanism remains polaronic.^30^ More telling is the comparison with bismuth-based analogs: Cs_3_Bi_2_I_9_ (0D) displays negative photoconductivity due to light-induced trap states and a moderate activation energy,^16^ while Rb_3_Bi_2_I_9_ adopting a 2D layered structure shows markedly lower Ea (0.135 eV) and non-overlapping small polaron tunneling (NSPT), enabled by enhanced inter-octahedral connectivity and lighter Rb^+^ mass facilitating ion mobility. These comparisons underscore a clear structure–property trend: dimensionality and halide identity critically govern carrier delocalization and activation barriers. For thermoelectric applications, Cs_3_Fe_2_Cl_9_'s high *E*_a_ may limit electrical conductivity (*σ*), yet its 0D framework, heavy Cl^−^ anions, and strong spin–lattice coupling are expected to severely suppress lattice thermal conductivity (*κ*_l_) a key requirement for high thermoelectric figure of merit (*ZT* = *σS*^2^*T*/*κ*). Moreover, the d^5^ electronic structure may enhance the Seebeck coefficient (*S*) through entropy-driven carrier filtering. Thus, despite its modest *σ*, Cs_3_Fe_2_Cl_9_ emerges as a promising candidate for mid-temperature thermoelectric, where ultralow *κ* and earth-abundant, non-toxic constituents outweigh moderate electrical performance especially when contrasted with higher-conductivity but potentially higher *κ* 2D analogs like Rb_3_Bi_2_I_9_. Future work should focus on measuring *κ* and *S* directly, and exploring nanostructuring or Cl/Br alloying to optimize the *σ*/*κ* balance.

## 8 Conclusion

Cs_3_Fe_2_Cl_9_ crystallizes in a zero-dimensional hexagonal structure (space group *P*6_3_/*mmc*) composed of isolated [Fe_2_Cl_9_]^3−^ bioctahedral dimers, which impart strong carrier localization and suppress long-range electronic coupling. Its wide indirect band gap of ∼2.98 eV and strong UV absorption make it unsuitable for visible-light photovoltaics but highly promising for deep-UV photodetection and radiation-sensitive applications. Despite a relatively high Urbach energy (0.98 eV) suggesting significant structural disorder, the material exhibits thermally activated charge transport governed by single small polaron hopping with an activation energy of 1.38 eV. This conduction mechanism, combined with high dielectric permittivity at low frequencies and robust structural stability, positions Cs_3_Fe_2_Cl_9_ as a compelling lead-free candidate for specialized optoelectronic and sensor technologies particularly where UV selectivity, radiation hardness, and environmental sustainability are prioritized over visible-light harvesting. While this study focuses on elevated-temperature transport (393–453 K), future work will explore low-temperature magnetic and optical behavior to fully assess its potential in cryogenic radiation detection.

## Conflicts of interest

The authors declare no competing interests.

## Data Availability

All data supporting the findings of this study are included with in the manuscript.
